# Prevalence and Predictors of Liver Fibrosis in People Living with Hepatitis B in Senegal

**DOI:** 10.3390/v14081614

**Published:** 2022-07-24

**Authors:** Adrià Ramírez Mena, Ndeye Fatou Ngom, Judicaël Tine, Kine Ndiaye, Louise Fortes, Ousseynou Ndiaye, Maguette Fall, Assietou Gaye, Daye Ka, Moussa Seydi, Gilles Wandeler, on behalf of the SEN-B Study Group

**Affiliations:** 1 Department of Infectious Diseases, Bern University Hospital, University of Bern, 3010 Bern, Switzerland; gilles.wandeler@insel.ch; 2 Graduate School of Health Sciences, University of Bern, 3010 Bern, Switzerland; 3 Service de Maladies Infectieuses et Tropicales, Fann University Hospital, Dakar 10700, Senegal; judicaelmalicktine@gmail.com (J.T.); louisefortes@yahoo.fr (L.F.); maguifall4@gmail.com (M.F.); dayeka10@gmail.com (D.K.); seydi.moussa@gmail.com (M.S.); 4 Centre de Traitement Ambulatoire de Fann, University Hospital Fann, Dakar 10700, Senegal; ndeyetouti98@gmail.com (N.F.N.); nekita1248@yahoo.fr (K.N.); assietougaye.diack@gmail.com (A.G.); 5 Centre Régional de Recherche et Formation Clinique à la Prise en Charge de Fann, Fann University Hospital, Dakar 10700, Senegal; weuzndiaye@yahoo.fr; 6 Institute of Social and Preventive Medicine, University of Bern, 3010 Bern, Switzerland

**Keywords:** hepatitis B, liver fibrosis, HIV, hepatitis B viral load, Senegal

## Abstract

Hepatitis B virus (HBV) infection is the first cause of liver cirrhosis and cancer in West Africa. Although the exposure to additional environmental and infectious risk factors may lead to the faster progression of liver disease, few large-scale studies have evaluated the determinants of HBV-related liver fibrosis in the region. We used transient elastography to evaluate the prevalence of liver fibrosis and assessed the association between HBV markers and significant liver fibrosis in a cohort of people living with HBV in Dakar, Senegal. The prevalence of significant liver fibrosis was 12.5% (95% confidence interval [CI] 9.6%–15.9%) among 471 people with HBV mono-infection (pwHBV) and 6.4% (95% CI 2.6%–12.7%) in 110 people with HIV/HBV co-infection (pwHIV/HBV) on tenofovir-containing antiretroviral therapy (*p* = 0.07). An HBV viral load > 2000 IU/mL was found in 133 (28.3%) pwHBV and 5 (4.7%) pwHIV/HBV, and was associated with significant liver fibrosis (adjusted odds ratio (aOR) 1.95, 95% CI 1.04–3.66). Male participants (aOR 4.32, 95% CI 2.01–8.96) and those with elevated ALT (aOR 4.32, 95% CI 2.01–8.96) were especially at risk of having significant liver fibrosis. Our study shows that people with an HBV viral load above 2000 IU/mL have a two-fold increase in the risk of liver fibrosis and may have to be considered for antiviral therapy, independent of other disease parameters.

## 1. Introduction

Hepatitis B virus (HBV) infection affects >10% of the general population in West Africa, and is the first cause of liver cirrhosis and cancer [1,2]. According to a recent meta-analysis, between 4% and 13% of people living with HBV in Africa had signs of liver cirrhosis, an indication for the immediate initiation of antiviral therapy [3]. The risk of liver-related complications in the region is further exacerbated by co-existing environmental factors, such as the exposure to aflatoxin, and co-infections with HIV, hepatitis delta or Schistosoma mansoni [4,5,6]. Although antiviral therapy effectively suppresses the HBV viral load and reduces the risk of HBV-related complications, such as the progression to liver cirrhosis and hepatocellular carcinoma (HCC), the criteria for its initiation in Africa are still debated [7,8].

The World Health Organization recently called for the elimination of viral hepatitis as a public health problem by 2030, which includes the reduction of liver-related mortality by 65% [9]. Understanding the natural history of the infection and the key drivers of HBV-related liver fibrosis in highly-endemic regions is key to meeting this objective. The REVEAL study demonstrated the strong association between the level of HBV replication and the incidence of HCC in Asia [10]. Similarly, the risk of developing liver cirrhosis was linked to HBV viral load, even among the HBeAg-negative individuals without transaminase elevation [11]. On the African continent, most of the information available on HBV-related clinical outcomes comes from studies among people with HIV and HBV co-infection, who are generally receiving tenofovir-containing antiretroviral therapy. Thus, the link between the virological determinants of HBV infection and the manifestations of liver disease needs to be explored to guide recommendations on eligibility for antiviral therapy.

Taking advantage of the infrastructure of two large referral HIV clinics in Dakar, Senegal, we established a prospective cohort (SEN-B) of people living with HBV (pwHBV) and HIV/HBV (pwHIV/HBV), to study the determinants of HBV infection and functional cure. In this analysis, we aimed to evaluate the association between HBV replication and liver fibrosis in people living with HBV in Senegal.

## 2. Materials and Methods

We conducted a cross-sectional analysis within the Senegalese Hepatitis B Cohort study (SEN-B) in Dakar. Since October 2019, all of the individuals aged >16 years and presenting with a confirmed positive hepatitis B surface antigen (HBsAg) test result at the Infectious and Tropical Disease Service (SMIT) and the Ambulatory Treatment Centre (CTA) at Fann University Hospital, were invited to participate in SEN-B. SMIT and CTA are the largest referral centers for HIV care in Senegal, with >3000 people living with HIV (pwHIV) under regular follow-up. We also undertook the systematic HBsAg screening for all of the pwHIV in current follow-up at the SMIT/CTA clinics, and included individuals with HIV/HBV co-infection into SEN-B [12]. The SEN-B cohort was approved by the national Ethics Committee, and all of the patients signed an informed consent to participate in the study.

The demographic, clinical and laboratory data, as well as transient elastography and abdominal ultrasound measurements, were collected at enrolment (baseline) and 6-monthly thereafter. The data were collected using standardized forms and were subsequently entered into an electronic database (ARPEGE^®^). The HBV surface antigens (HBsAg) were tested, using a lateral-flow assay test (NovaTest^®^|Atlaslink-inc ISO 13485) and confirmed with the e411 cobas^®^ HBsAg II (2011–08 V.10) platform. The HBV DNA levels were measured using the cobas^®^/TaqMan^®^ (Roche Diagnostic Systems, Meylan, France) with a threshold of detection of 20 IU/mL. HIV, hepatitis C virus (HCV) and hepatitis D were tested using commercial lateral-flow assays (Determine^®^ HIV/HCV, Abbot, and Diasorin^®^ for HDV). The alanine aminotransferase (ALT) elevation was defined as >40 IU.

Liver stiffness was measured by transient elastography (TE) during clinic visits by a single trained operator, according to the instructions provided by the manufacturer (Fibroscan^®^, Echosens, France). The liver stiffness measurements (LSM) were considered valid only if 10 successful acquisitions were obtained, and the interquartile range (IQR) to median ratio of the 10 acquisitions was <0.3 [13]. We used an M probe for standard assessment and a XL probe if BMI was >25 kg/m^2^. The cut-off value of 7.0 kPa was used to estimate the number of subjects with significant fibrosis, and 11.0 kPa for cirrhosis [14]. Alcohol consumption was assessed using the Alcohol Use Disorders Identification Test-Consumption (AUDIT-C), modified to measure drinking in the prior 6 months [15,16]. Current abstinence was an AUDIT-C of 0 points, moderate drinking was one–two points for women and one–three for men, and unhealthy consumption was >two for women and >three for men.

### Statistical Analysis

We compared the demographic and clinical characteristics between pwHBV and pwHIV/HBV at enrolment into the cohort using descriptive analyses. The *χ*^2^ test was used for the categorical variables and the Wilcoxon rank-sum test for the continuous ones. We evaluated the proportion of the participants in the different liver stiffness groups (no fibrosis, fibrosis, cirrhosis) by HIV status, sex, ALT and HBV DNA levels, and compared the estimates between pwHBV and pwHIV/HBV using Fisher’s exact test. We used multivariable logistic regression to evaluate the association between HBV viral load and significant fibrosis, after adjustment for the following baseline variables: age; sex; body mass index (BMI); use of traditional medicine; unhealthy alcohol consumption; HIV status and ALT values. The association was further explored by repeating the analyses after excluding (i) pwHIV/HBV and (ii) pwHBV who had received HBV antiviral treatment prior to the outcome measurement.

Since the individuals with elevated ALT levels tend to have higher LSM than those with normal ALT levels at the same stage of liver fibrosis, we performed a sensitivity analysis, adapting TE cut-offs based on levels of ALT [17]. For the individuals with elevated ALT (>40 IU) we used TE cut-off values >7.5 kPa for significant fibrosis and >12.0 kPa for cirrhosis. All of the statistical analysis were completed using Stata 16.1 (Stata corp.) and the regression plots were produced by user command “coefplot” and “grstyle” [18,19].

## 3. Results

### 3.1. Characteristics of Study Population

We included 581 individuals with a positive HBsAg test, of whom 110 (18.9%) were living with HIV. The median age at enrolment was 31 years (interquartile range (IQR) 25–39) among pwHBV and 46 years (IQR 38–54) in pwHIV/HBV (*p* < 0.001, Table 1). The median BMI as well as the proportion of women were similar in both of the groups. Unhealthy alcohol consumption was infrequent: 1.9% in pwHBV and 2.7% in pwHIV/HBV. Only two of the participants had antibodies against hepatitis C virus (HCV) infection, and three against hepatitis D virus (HDV) infection.

All of the pwHIV/HBV were receiving TDF as part of their ART regimen, for a median time of 5.3 years (IQR; 3.0–8.4), while only 4.7% of the pwHBV were on antiviral therapy at the time of inclusion, for a median time of 0.6 years (IQR 0–1.4). More of the pwHIV/HBV presented with elevated ALT measurements (>40 IU) compared to the pwHBV (17.3% vs. 7.8%; *p* = 0.01). HBV DNA was <20 IU in 76/471 (16.2%) of the pwHBV and in 89/110 (83.2%) of the pwHIV/HBV (*p* < 0.001). A higher proportion of the pwHBV presented with HBV DNA >2000 IU/mL compared to the pwHIV/HBV (28.3% vs. 4.7%; *p* < 0.001). A summary of the characteristics of the study population is presented in Table 1.

### 3.2. Prevalence of Significant Liver Fibrosis and Cirrhosis

The prevalence of significant liver fibrosis was 12.5% (95% CI 9.6–15.9) in the pwHBV and 6.4% (95% CI 2.6–12.7) in the pwHIV/HBV (*p* = 0.07), whereas the estimate for liver cirrhosis was similar in both of the groups (pwHBV: 4.0%, 95% CI 2.4–6.0; pwHIV/HBV: 3.6%, 95% CI 1.0–9.0, *p* = 1.00). Among the treatment-naïve pwHBV (*n* = 449), the prevalence of significant liver fibrosis was 12.2% (95% CI 9.3–15.6) and cirrhosis 3.6% (95% CI 2.1–5.8).

**Figure 1:**
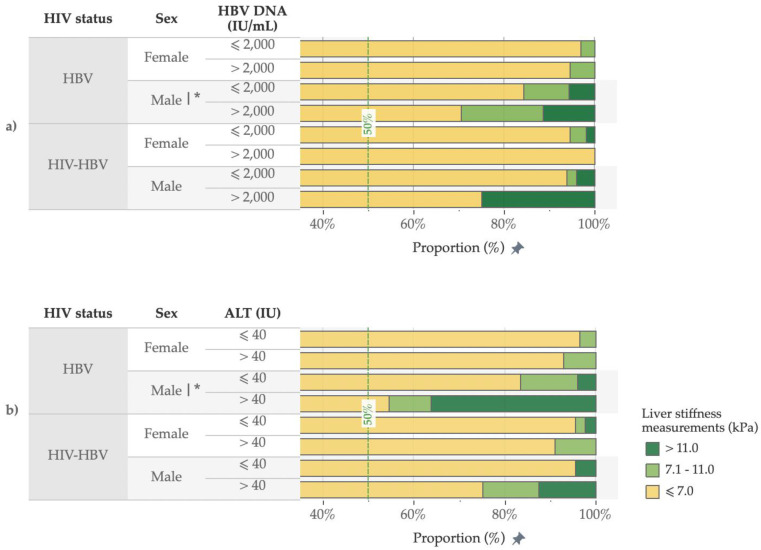
Proportion of participants in the different liver stiffness measurement categories, by HIV status, sex, HBV DNA (**a**) and ALT (**b**) values. |* *p* < 0.05 in univariable analyses.

The distribution of LSM categories by HIV status, sex, ALT and HBV DNA values is presented in Figure 1. In the pwHBV, men were more likely to have significant fibrosis (19.9% vs. 3.7%, *p* < 0.001) and cirrhosis (7.4% vs. 0.0%, *p* < 0.001, Table S1, Supplementary Materials) compared to women. Furthermore, the pwHBV with HBV DNA >2000 IU/mL were more likely to have significant fibrosis compared to those with HBV DNA ≤ 2000 IU/mL (male: 29.5% vs. 15.8%, *p* = 0.01; female: 5.5% vs. 3.1%, *p* = 0.43). Likewise, male pwHBV with elevated ALT (> 40 IU) were more likely to present with significant fibrosis compared to those with normal ALT levels (45.4% vs. 16.6%, *p* = 0.001). Although the numbers were small, we found similar associations between HBV DNA or ALT and liver fibrosis among male pwHIV/HBV (Table S1, Supplementary Materials). In the secondary analyses, which focused on the treatment-naïve pwHBV, the differences in the proportions of participants with fibrosis and cirrhosis between HBV DNA and ALT groups were similar to those from the main analyses (Table S2, Supplementary Materials).

### 3.3. Risk Factors for Significant Fibrosis and Cirrhosis

**Figure 2:**
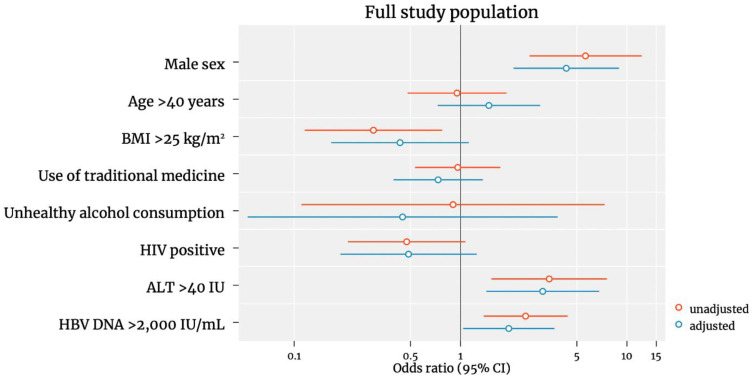
Predictors of significant liver fibrosis in the full study population. Odds ratios were plotted on a logscale.

In multivariable analyses including all of the participants, those with HBV DNA > 2000 IU/mL were more likely to have significant fibrosis than those with low levels of HBV DNA (adjusted odds ratio (aOR) 1.95, 95% CI 1.04–3.66, *p* = 0.04) (Figure 2). 

**Figure 3:**
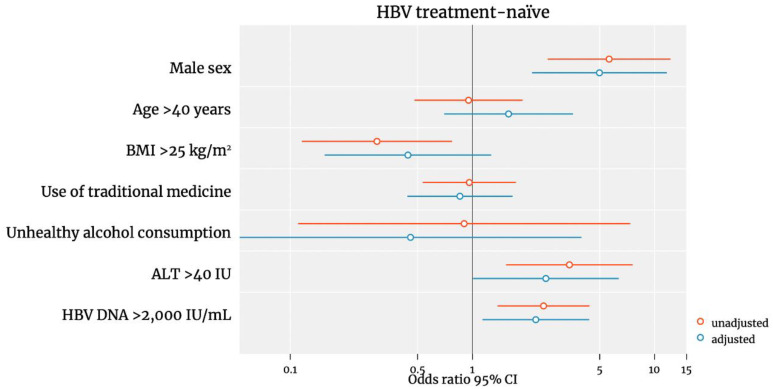
Predictors of significant liver fibrosis among treatment-naïve pwHBV. Odds ratios were plotted on a logscale.

Male sex (aOR 4.32, 2.01–8.96, *p* < 0.001) and elevated ALT (aOR 4.32, 2.01–8.96, *p* < 0.001) were also independent risk factors of significant fibrosis. However, HIV/HBV co-infection, age > 40 years, use of traditional medicine and hazardous alcohol consumption were not associated with liver fibrosis. Being overweight (BMI > 25) was associated with a reduced risk of significant fibrosis in the unadjusted analysis, however this association did not remain significant in the adjusted analysis (aOR 0.43, 95% CI 0.16–1.12; *p* = 0.09). We additionally performed an analysis including only the untreated pwHBV and the same factors remained associated with liver fibrosis (Figure 3). Due to the low number of cirrhosis cases (22 in male and 1 in female|19 in pwHBV and 4 in pwHIV/HBV), our analysis was underpowered to estimate the associations with cirrhosis. 

Our results were similar in the sensitivity analysis using the definitions of significant fibrosis and cirrhosis adapted to ALT measurements (Figure S1, Supplementary Materials). Male sex (aOR 4.80, 95% CI 2.24–10.31; *p* < 0.001), elevated HBV DNA > 2000 IU/mL (aOR 3.22, 95% CI 1.46–7.09; *p* = 0.004) and elevated ALT (aOR 3.22, 95% CI 1.46–7.09; *p* = 0.004) remained associated with higher odds of significant fibrosis.

## 4. Discussion

In our cross-sectional assessment of the people living with HBV in urban Senegal, 12% of the treatment-naïve people living with HBV and 6% of those living with HIV and HBV on ART had significant liver fibrosis. The participants with an HBV viral load >2000 IU/mL were twice as likely to present with significant fibrosis as those with lower HBV DNA levels. Male sex and elevated ALT were additional independent risk factors for liver fibrosis. Our findings suggest a strong association between HBV replication and liver fibrosis in Senegal, and, if confirmed in prospective cohort studies in the region, they may argue for the early initiation of antiviral therapy in the context of HBV replication, to reduce the risk of development of liver disease.

Although transient elastography is considered the best method for the non-invasive measurement of liver fibrosis in individuals with HBV infection, it is rarely available in African healthcare-settings. A recent meta-analysis, including both pwHBV and pwHIV/HBV, found only seven studies across Africa reporting fibrosis using TE [3]. The pooled prevalence of significant fibrosis was 11.6%, and the estimate for cirrhosis was 6.1%, both very similar to our results. In contrast, Aberra et al. found a prevalence of fibrosis reaching 26% in a large tertiary care referral center in Ethiopia, highlighting the importance of considering the type of healthcare setting in the interpretation of the HBV-related liver complications in sub-Saharan Africa [20]. Although our participants were enrolled into the SEN-B cohort in a tertiary care center, our study population can be considered to represent the general population, as >80% were referred from community/primary care structures after routine testing at a transfusion center, patients’ association or general practitioner’s office in the absence of signs of liver disease.

Our findings highlight the strong association between HBV viral replication and significant liver fibrosis in pwHBV from sub-Saharan Africa. Two landmark studies from the REVEAL-HBV cohort identified HBV DNA levels as the most important predictor for the progression to end-stage liver disease [10,11]. These two analyses of the pwHBV in Taiwan showed that HBV DNA levels at enrolment was an independent predictor of cirrhosis, HCC and all-cause mortality. Importantly, the same observation was made when considering only the HBeAg-negative individuals with normal ALT values [11]. In one of the few studies from Africa, HBV viremia was also found to be linked with an increased risk of cirrhosis and HCC [21]. In our study, elevated HBV DNA was independently associated with liver fibrosis, including in the subset of treatment-naive pwHBV and after adjustment for ALT values. If the prospective studies show a similar link between elevated DNA levels and the development of liver fibrosis, the presence of HBV replication could become a strong argument for the early initiation of antiviral therapy, independent of other markers of activity. In a recently published study of >80,000 individuals with chronic HBV infection in the USA, Wong et al. suggested a simplified stepwise strategy to increase eligibility for antiviral therapy and estimated that treating individuals with a viral load >2000 IU/mL, regardless of ALT values, would increase the proportion of treatment eligible individuals by 34% [22]. To progress towards hepatitis B elimination in Africa, simplified treatment criteria, using HBV replication as the central criterion, may be crucial to overcome the barriers to treatment and reduce the burden of liver disease in pwHBV [7,22].

Male sex was associated with a five-fold increased risk of significant fibrosis, and this association remained similar when considering only untreated pwHBV. Similar results were observed in other studies, using TE to measure liver fibrosis among Asian populations with HBV [23,24]. It has been suggested that female sex hormones could play a protective role for liver fibrosis, but this hypothesis has yet to be evaluated in African populations [25]. In contrast to the existing literature, we did not find an association between HIV status and an increased risk of liver fibrosis. However, most of the previous studies showed HIV co-infection to be linked to elevated LSM before ART was initiated, and limited data exist on the treatment-experienced populations [26,27,28,29]. In prospective studies from Tanzania and Zambia, liver fibrosis regression occurred during ART, irrespective of HBV infection [27,30]. The long-term follow-up of cohorts, including pwHBV with and without HIV-coinfection, will be crucial to understand the impact of HIV infection on liver disease progression.

Our study is among the first in sub-Saharan Africa to assess the determinants of liver fibrosis using TE in people with different degrees of HBV replication. However, despite the high diagnostic accuracy of TE for the detection of liver fibrosis in pwHBV [31], optimal thresholds are yet to be determined for HIV/HBV co-infection and in the presence of liver inflammation. Nevertheless, our results remained similar across sensitivity analyses, including after adjustment of the TE thresholds according to the ALT levels. We had incomplete data on HDV co-infection for half of the study participants, which may have under/over-estimated the prevalence. Furthermore, we could not account for other liver fibrosis risk factors, such as Schistosomiasis sp. infection, which is highly endemic in Senegal. However, its association with liver fibrosis is still a matter of debate in individuals with HBV infection [32].

## 5. Conclusions

Our findings from a large cohort of people living with HBV in Senegal suggest a strong association between HBV replication and liver fibrosis. Considering antiviral therapy eligibility based on HBV DNA levels independently of other factors may help reduce the burden of liver fibrosis among people with HBV infection in sub-Saharan Africa, should our results be confirmed in prospective studies.

## Figures and Tables

**Table 1:** Characteristics of the study population at the time of liver fibrosis assessment, by HIV co-infection status.

Characteristics	Persons Living with HBV (*n* = 471)	Persons Living with HIV/HBV (*n* = 110)
Male sex, *n* (%)	256 (54.4)	52 (47.7)
Median age in years (IQR) *	31 (25–39)	46 (38–54)
BMI > 25 kg/m^2^, *n* (%)	109 (24.4)	24 (23.5)
Use of traditional medicine, *n* (%) *	173 (36.7)	28 (25.7)
Any alcohol consumption, *n* (%)	23 (4.9)	6 (5.5)
Unhealthy alcohol consumption, *n* (%)	9 (1.9)	3 (2.7)
Smoker, *n* (%) *	9 (1.9)	8 (7.3)
HCV Ab-positive, *n* (%)	2 (0.8)	0 (0)
HDV Ab-positive ^1^, *n* (%)	1 (0.6)	2 (2)
TDF-experienced, *n* (%)	22 (4.7)	100 (100)
Median ALT in IU (IQR) *	17 (12–24)	19 (13–34)
ALT > 40 (IU), *n* (%) *	36 (7.8)	19 (17.3)
Median HBV DNA in IU/mL (IQR) *	547.5 (92–2600)	0 (0)
HBV DNA undetectable, *n* (%) *	76 (16.2)	89 (83.2)
HBV DNA > 2000 (IU/mL), *n* (%) *	133 (28.3)	5 (4.7)

* *p* < 0.05 in univariable analysis. ^1^ Test results available for 272 individuals only.

## Data Availability

The data presented in this study are available on request from the corresponding author.

## Supplementary Materials

Table S1. Proportions of participants with significant fibrosis and cirrhosis among pwHIV/HBV by sex, ALT and HBV DNA values. Table S2. Proportions of participants with significant fibrosis and cirrhosis among untreated pwHBV by sex, ALT and HBV DNA values. Figure S1. Predictors of significant liver fibrosis using adapted liver stiffness measurments thresholds to ALT levels in the full study population. Odds ratios were plotted on a logscale. The following supporting information can be downloaded at: https://www.mdpi.com/article/10.3390/v14081614/s1.
