# Supplementary material for: Prevalence and Predictors of Liver Fibrosis in People Living with Hepatitis B in Senegal
Source: Viruses. 2022 Jul 24;14(8):1614. doi: 10.3390/v14081614 (PMC9331503; doi:10.3390/v14081614)
Supplement: Supplementary file 1 [file viruses-14-01614-s001.zip › viruses-1783192-supplementary.pdf]

Supplementary Materials:

Table S1. Proportions of participants with significant fibrosis and cirrhosis among pwHIV/HBV by sex , ALT and HBV DNA values.

**Liver fibrosis**	**Male**			**Female**		
	**HBV DNA ⚜2000 IU/ml**	**HBV DNA >2000 IU/ml**	***p value***	**HBV DNA ⚜2000 IU/ml**	**HBV DNA >2000 IU/ml**	***p value***
Significant fibrosis, n(%)	3 (6.3)	1 (25.0)	.281	3 (5.6)	0 (0)	.945
Cirrhosis, n(%)	2 (4.2)	1 (25.4)	.217	1 (1.9)	0 (0)	1.0

**Liver fibrosis**	**Male**			**Female**		
	**ALT ⚜ 40 IU**	**ALT > 40 IU**	***p value***	**ALT ⚜ 40 IU**	**ALT > 40 IU**	***p value***
Significant fibrosis, n(%)	2 (4.6)	2 (25.0)	.107	2 (4.3)	1 (9.1)	.474
Cirrhosis, n(%)	2 (4.6)	1 (12.5)	.401	1 (2.1)	0 (0)	1.0

Abbreviations: HBV: Hepatitis B virus; DNA: Desoxyribonucleic Acid; ALT: Alanine aminotransferase; IU: International Units.

Table S2. Proportions of participants with significant fibrosis and cirrhosis among untreated pwHBV by sex , ALT and HBV DNA values.

.

**Liver fibrosis**	**Male**			**Female**		
	**HBV DNA ⚜2000 IU/ml**	**HBV DNA >2000 IU/ml**	***p value***	**HBV DNA ⚜2000 IU/ml**	**HBV DNA >2000 IU/ml**	***p value***
Significant fibrosis, n(%)	25 (14.8)	22 (29.9)	.014	5 (3.3)	3 (5.8)	.425
Cirrhosis, n(%)	8 (4.7)	8 (10.5)	.100	0 (0)	0 (0)	_

**Liver fibrosis**	**Male**			**Female**		
	**ALT ⚜ 40 IU**	**ALT > 40 IU**	***p value***	**ALT ⚜ 40 IU**	**ALT > 40 IU**	***p value***
Significant fibrosis, n(%)	36 (16.3)	9 (42.9)	.006	7 (3.8)	1 (7.1)	.450
Cirrhosis, n(%)	8 (3.6)	7 (33.3)	<.001	0 (0)	0 (0)	_

Abbreviations: HBV: Hepatitis B virus; DNA: Desoxyribonucleic Acid; ALT: Alanine aminotransferase; IU: International Units

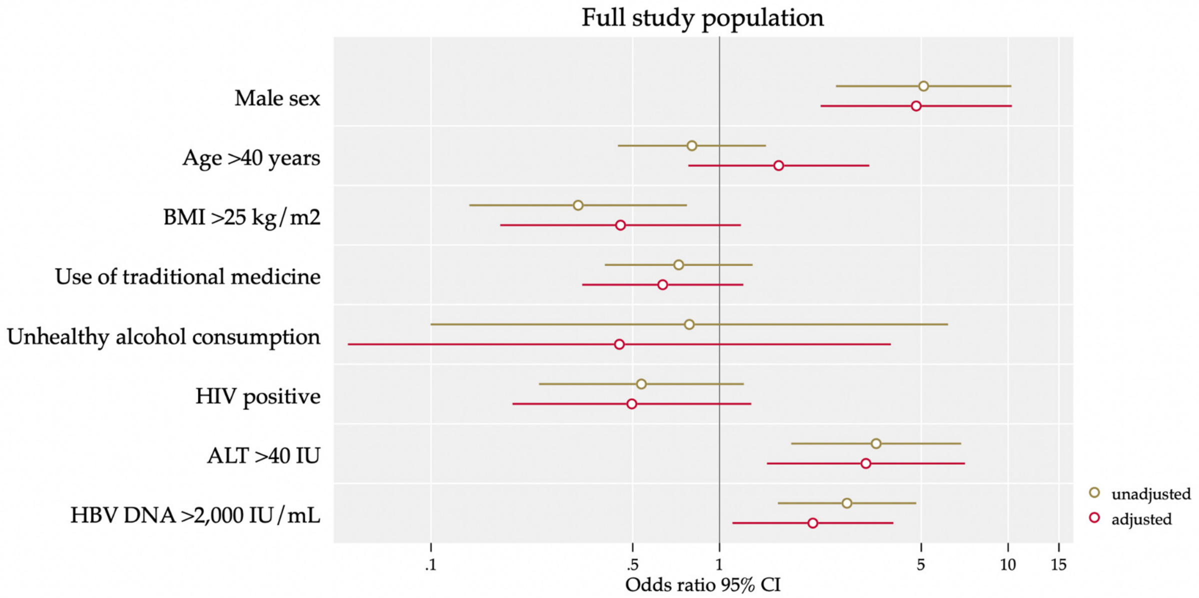

Figure S1. Predictors of significant liver fibrosis using adapted liver stiffness measurments thresholds to ALT levels in the full study population. Odds ratios were plotted on a logscale.

Abbreviations: BMI: Body mass index; HIV: Human immunodeficiency virus; HBV: Hepatitis B virus; DNA: desoxyribonucleic acid; ALT: Alanine aminotransferase; IU: International units
